# Adrenal Cavernous Hemangioma: A Rarely Perceived Pathology—Case Illustration and Review of Literature

**DOI:** 10.1155/2019/8463890

**Published:** 2019-12-17

**Authors:** Jad A. Degheili, Nassib F. Abou Heidar, Mouhammad El-Moussawi, Ayman Tawil, Rami W. Nasr

**Affiliations:** ^1^Department of Surgery, Division of Urology, American University of Beirut-Medical Center, Beirut 1107 2020, Lebanon; ^2^Department of Pathology and Laboratory Medicine, American University of Beirut-Medical Center, Beirut 1107 2020, Lebanon

## Abstract

Cavernous hemangiomas are endothelial tumors that rarely affect the adrenal glands. Most of these tumors remain silent and are incidentally found on abdominal imaging. Hardly ever, these tumors are endocrinologically functional. They may present as vague abdominal pain. Surgical resection remains the mainstay for large masses. In this paper, we are presenting a case of adrenal cavernous hemangioma in a 83-year-old male patient who initially presented for workup of vague abdominal and bilateral flank pain. A computed tomography scan of the abdomen showed an 8 cm right adrenal adenoma which was metabolically nonfunctional. The mass was completely resected through an open subcostal incision, with no encountered postoperative complications. A highlight of all published cases of adrenal hemangiomas since 1955 is also presented and reviewed.

## 1. Introduction

Incidental adrenal masses are a growing concern, especially with the significant increase in their detection upon the many abdominal imaging modalities utilized for the workup of various patient's complaints. The prevalence of incidental adrenal masses approaches 7% in the general population [1]. Adrenal masses tend to be heterogeneous in nature and comprise of benign adenomas, secreting adenomas, lymphomas, myelolipomas, cysts, and adrenocortical carcinoma most commonly as well as other rarer pathologies such as adenomatoid tumors and sex-cord stromal tumors [2]. On the other hand, adrenal cavernous hemangiomas are unusual tumors arising from the endothelial lining of blood vessels [3]. We hereby present a case of a cavernous hemangioma diagnosed on histopathology after an adrenalectomy, as well as a review of all reported cases of this entity in the literature.

## 2. Case Presentation

A 83-year-old previously healthy male presented with vague abdominal and bilateral flank pain of several months duration. The pain was dull in nature, with no recent change in weight and appetite, no reported hematuria, no gastrointestinal symptoms, and no reported episodes of headache. All basic blood work up, including complete blood count, creatinine, electrolytes, and liver function tests, were within normal range. An initial imaging with an enhanced computed tomography (CT) scan of the abdomen and pelvis was performed, revealing a right supra renal mass measuring around 8 cm in greatest dimension, possessing a significant enhancement, with a Hounsfield Unit (HU) of 15 on noncontrast phase and a 55HU on the contrast phase. No other abdominal or pelvic findings were noted.

**Figure 1:**
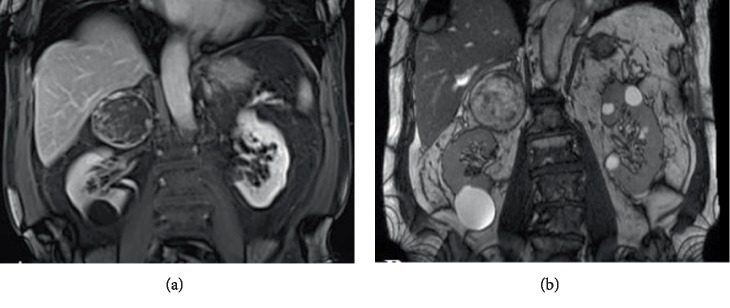
Enhanced magnetic resonance images (MRI) of the abdomen and pelvis with gadolinium. (a) Coronal view of an in-phase T1-weighted image identifying a large suprarenal mass measuring around 7.3 × 6.5 × 6 cm occupying the space of the right adrenal gland, showing a significant rim enhancement with marked vascularity. (b) Coronal view of a T2-weighted image identifying the similar right suprarenal mass with heterogeneous component inside it, possessing various signal intensities.

For better characterization of such adrenal lesion, a Magnetic Resonance Imaging (MRI) of the abdomen with gadolinium was requested. Again showing was a mass in the right suprarenal space, measuring 7.3 × 6.5 × 6 cm, showing heterogeneous signal intensity on all sequences, predominately mildly increased on T2-weighted and predominately decreased signal on T1-weighted images, and no appreciable signal drop on the out-of-phase images. The lesion appeared inseparable from the lateral limb of the right adrenal gland and remained separate from the upper edge cortex of the right kidney (Figure 1).

Imaging was also accompanied by a full metabolic work-up, as usually performed for any incidentally discovered large adrenal mass, including: urine and plasma metanephrines, dexamethasone suppression test, DHEA-S, and aldosterone/renin ratio, and all yielded results were within normal range.

Due to the size of the tumor, a shared decision was made to surgically remove the adrenal gland due to a potential risk of being an adrenocortical carcinoma. The patient therefore underwent a right radical adrenalectomy through an open subcostal incision. Intraoperatively, the adrenal was noticeably friable and tended to bleed on minimal manipulation, which was expected from the preoperative imaging done (Figure 1). No significant blood loss was encountered since complete dissection of the adrenal gland was done for better hemostatic control.

**Figure 2:**
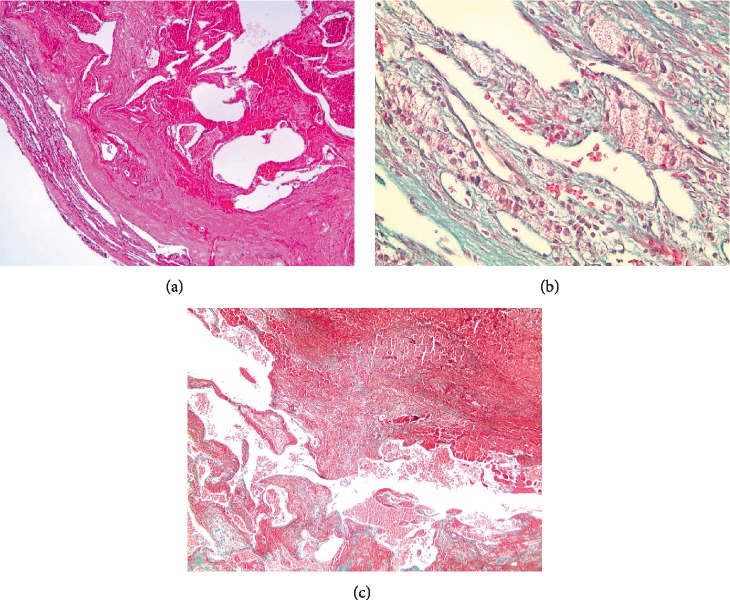
Histopathological images of an adrenal cavernous hemangioma cross section, showing dilated capillaries with capsule and significant fibrosis on Hematoxylin and Eosin (H&E) staining (a). Magnified image showing red blood cells inside dilated capillaries (b), divided by thick fibrous septa (c).

On gross pathological examination, the specimen measured 8 × 7 × 3 cm and the tumor was shiny tan-yellow, in nature with distinct demarcation from the normal adrenal parenchyma. Histologically, the tumor was proven to be a hemorrhagic cavernous hemangioma (Figure 2).

Patient recovered well after his operation with no complications encountered thereafter. He was discharged home on his fourth postoperative day, to follow-up in clinic one month from discharge.

## 3. Discussion

Cavernous hemangiomas are unusual tumors of the endothelial linings with a propensity for skin, liver, and brain involvement [3]. Cavernous hemangiomas tend rarely to affect the genitourinary system [3].

Adrenal hemangiomas are one of the rarest nonfunctioning benign adrenal tumors that are commonly diagnosed postoperatively [4]. Although many cases of adrenal hemangiomas were presented at autopsy reports before 1869, the first surgical adrenal hemangioma was reported by Johnson and Jeppesen in 1995 [3].

Sixty-six cases of adrenal cavernous hemangiomas were published between the years 1955 and 2018 (Table 1), and identified after conducting an extensive literature review using PubMed, Medline, Embase, and Scopus databases. These cases were reviewed and summarized in Table 2. The median age of patients at diagnosis was 60 years. This neoplasm had a female preponderance with a female to male prevalence ratio of 3 to 2. No laterality preference was associated with adrenal hemangiomas. Two bilateral cases were only reported in the literature so far. Metabolic workup of adrenal neoplasms was normal in 45 of the 66 reported cases. Only 6 clinically functional adrenal hemangiomas were identified; with 3 cases of hyperaldosteronism and three other cases of subclinical Cushing's syndrome. The so far reported cases of adrenal hemangiomas, with detailed published tumor characteristics, exhibited a mean diameter of 11 cm and a mean weight of 752 grams. Of the 66 published cases, 38 were incidentalomas that were clinically silent and asymptomatic; 8 presented with vague abdominal symptoms such as bloating, epigastric pain, and heaviness, and 6 cases reported solely flank pain. Another two cases presented with spontaneous rupture of the adrenal mass with subsequent retroperitoneal hemorrhage and hematoma; a serious complication that is seldom seen.

On imaging, 32 adrenal masses were associated with speckled calcification, a historically described finding in any adrenal hemangioma; and 29 cases failed to show calcifications. Sixty-five of the reported cases were managed surgically; out of them, 47 were excised through an open approach, and the remaining 16 cases were excised laparoscopically.

Most of the cavernous hemangiomas reported in the literature are incidental findings on imaging performed for unrelated or unspecific complaints [66]. These tumors grow insidiously until they reach a large size and start producing symptoms by virtue of mass effect and mechanical pressure on adjacent organs. Vague symptoms such as fever, weight loss, and sweating are nonspecific findings for neoplastic lesions that are reported in adrenal hemangiomas [41, 67]. Flank pain in the setting of normal urine analysis is the most commonly reported presenting complaint in symptomatic patients. Hypertension has been identified as a presenting symptom for adrenal hemangiomas in the setting of normal adrenal functions. Six cases reported so far presented with a hyperfunctioning adrenal mass; three of them presented with signs of hyperaldosteronism such as hypokalemia, and three other cases were consistent with subclinical Cushing [20, 44].

Histopathologically, adrenal hemangiomas are stratified into two subtypes: cavernous and capillary. The cavernous subtype is composed of an enlarged mass of blood filled endothelially-lined sinusoids, displacing potentially normal tissues. Whereas in the rarer capillary subtype, it is composed of small tufts of submucosal capillaries arranged in radiating loops or lobules [20].

Historically, adrenal hemangiomas were usually identified on plain abdominal radiographies for unrelated complaints. On radiographs, these neoplasms appear as calcified masses. Calcifications, if present, are universally speckled through the entire mass as opposed to the curvilinear calcifications usually associated with adrenal pseudocysts [68]. Computed tomography can effectively define the anatomy, configuration, and volume of any adrenal mass and can partially delineate the general tissue's characteristics. On CT scanning, these masses are generally encapsulated and heterogenous with scattered calcifications [68]. Calcifications are usually correlated with benign adrenal lesions; however, some reports describe calcifications in malignant lesions as well. Therefore, calcifications become an unreliable sign to assess the malignant potential of any adrenal mass. Cavernous hemangiomas are mostly masses with smooth margins and low relative attenuation coefficient [20, 68]. However, rim-like calcifications within the suprarenal glands have been adopted radiologically as a sign of benignity of such lesions. A radiologic sign was first described by Rothberg et al., referred to as phleboliths, which are round calcifications with translucent centers. This finding is considered pathognomonic for adrenal gland hemangiomas [68, 69]. CT scan has been shown to be superior to ultrasound for suprarenal masses. The masses are usually heterogeneously echogenic on ultrasonography. Magnetic Resonance Imaging (MRI) has sometimes been used, although a CT scan is enough as an imaging modality to identify adrenal neoplasms. Cavernous hemangiomas are hypo-intense masses on T1-weighted images and hyper-intense on T2-weighted images with peripheral enhancement after contrast administration [66].

Although not required for routine diagnostic workup of any adrenal masses, angiography on adrenal hemangiomas can reveal marked neovascularity with small vascular channels, usually arranged in a rim-like manner which retain contrast in delayed films [69]. These angiomas could be of many variants which include: angiomyelolipoma (more commonly seen), angiolipomas, cavernous hemangiomas, or epithelioid hemangioendothelioma, depending on the histopathological differences. Moreover, during pathologic examination, adrenal hemangiomas could be mistaken for adrenocortical carcinoma that has undergone cystic degeneration; therefore proper assessment of the subcapsular area is paramount [25].

After identification of adrenal masses on imaging, the common practice necessitated a full hormonal and metabolic workup to rule out primary functioning adrenal neoplasms mainly pheochromocytomas. Most cases of adrenal cavernous hemangiomas are nonsecretory and hormonally silent neoplasms [44]. Due to the scarcity of this condition, no guidelines have been developed so far to guide the treatment and therapeutic management of such entity.

Tumors originating from vessels could be associated with syndromes, but these are rather neonatal tumors, and not acquired tumors such as our present case. Nevertheless, there has been a previous single report of an adrenal cavernous hemangioma associated with familial adenomatous polyposis [57].

Most adrenal hemangiomas reported in the literature were managed surgically [20]. Asymptomatic small and benign-looking masses may be treated medically and conservatively with close monitoring. However, the follow-up schedule tends to be according to physician's preference. Larger masses bear the risk of spontaneous hemorrhage and should be resected surgically [20, 44]. Early cases were operated through open adrenalectomy. However, a laparoscopic approach is favored due to better postoperative results and lesser complications [70]. Knowing that, the risk of malignancy might sway the operating surgeon against a minimally invasive approach.

## 4. Conclusion

Adrenal cavernous hemangioma is a rare entity that might be encountered when dealing with an adrenal pathology. Surgical resection is sometimes necessary to rule out any malignant potential and alleviate symptoms secondary to mass effect. Retroperitoneal bleeding is a concern in such pathology, especially when large lesions are detected. Observation is an alternative in cases where lesions are small, asymptomatic, and metabolically inactive, especially when confirmed by biopsy.

## Figures and Tables

**Table 1:** List of all published cases of adrenal cavernous hemangiomas.

Case number	Authors (year of publication)	Age/gender	Laterality	Size (cm)	Presentation	Surgery
1	Johnson and Jeppesen (1955) [3]	46/F	Right	6.5 ∗ 4 ∗ 3	Hypertension	Open right adrenalectomy
2	Elliot et al. (1963) [5]	37/F	Left	25	Incidental finding	Open left adrenalectomy
3	Chodof et al. (1966) [6]	76/F	Left	18 ∗ 16	Abdominal mass and discomfort	Open left adrenalectomy
4	Weiss and Schulte (1966) [7]	70 /M	Right	11 ∗ 7 ∗ 6	Acute urinary retention (−ve met)	Open right adrenalectomy
5	Ruebel (1973) [8]	75/M	Right	8 ∗ 7.3 ∗ 6.5	Hematuria	Open right adrenalectomy
6	Rothberg et al. (1978) [9]	72`/F	Right	14 ∗ 10 ∗ 7	Long standing hypertension	Open right adrenalectomy
7	Rothberg et al. (1978) [9]	74/F	Left	9 ∗ 8	Incidental finding	Open left adrenalectomy
8	Vargas (1980) [10]	67/F	Left	NA	Incidental finding on barium study for chronic anemia	Open left adrenalectomy
9	Lee et al. (1982) [11]	59/F	Right	8.5 ∗ 7 ∗ 6	Incidental abdominal calcification	Open right adrenalectomy
10	Orringer et al. (1983) [4]	51/M	Right	17	Epigastric heaviness	Exploratory laparotomy with adrenalectomy
11	Goren et al. (1986) [12]	79/F	Right	9 ∗ 7 ∗ 5	Incidental finding (−ve metab)	Open right adrenalectomy
12	Nakagawa et al. (1986) [13]	71/M	Left	10 ∗ 18 ∗ 24	Night sweat and generalised fatigue	Open left adrenalectomy
13	Guerin et al. (1988) [14]	78/F	Left	3 ∗ 2.5 ∗ 2.5	Elevated ESR	Open left adrenalectomy
14	Derchi et al. (1989) [15]	69/F	Left	20	Incidental finding	Open left adrenalectomy
15	Derchi et al. (1989) [15]	60/M	Right	18	Hepatomegaly and abdominal pain	Open right adrenalectomy
16	Yoshihirio et al. (1990) [16]	78/F	Left	6 ∗ 5.5 ∗ 5	Incidental finding	Open left adrenallectomy
17	Honig et al. (1991) [17]	73/M	Left	NA	Incidental finding	Exprolatory laparotomy with adrenalectomy
18	Takahe et al. (1991) [18]	55/M	Left	10 ∗ 9 ∗ 9	Incidental finding	Open left adrenalectomy with splenectomy
19	Salup et al. (1992) [19]	73/F	Left	15	Syncope (incidental finding)	Open left adrenalectomy,distal pancreatectomy, splenectomy and left radical nephrectomy
20	Hamrick et al. (1993) [20]	66/M	left	9	Incidental finding	Open adrenalectomy
21	Sabanegh et al. (1993) [21]	60/F	left	20 ∗ 20	Incidental finding	Open left adrenalectomy
22	Boraschi et al. (1995) [22]	64/M	right	10 ∗ 8 ∗ 6.5	Megaloblastic anemia	Open adrenalectomy
23	Stumvoll et al. (1996) [23]	60/F	Right	8	Mineralocorticoid excess syndrome	Open partial right adrenalectomy
24	Marotti et al. (1997) [24]	68/F	Left	14 ∗ 10 ∗ 10	Incidental finding	Open adrenalectomy
25	Marotti et al. (1997) [24]	60/F	Left	9 ∗ 7.5 ∗ 5	Incidental finding	Open adrenalectomy
26	Oh et al. (1997) [25]	56/M	Right	6 ∗ 5 ∗ 4	Right flank discomfort	Open right adrenalectomy
27	Hayakawa et al. (1998) [26]	56/M	Left	5	Incidental finding	Open left adrenalectomy
28	Hisham et al. (1998) [27]	61/F	Right	25	Flank pain	Open right adrenalectomy
29	Makiyama et al. (1998) [28]	61/F	Right	5.5 ∗ 3.5 ∗ 3.5	Incidental finding	Open right adrenalectomy
30	Thiele and Bodie (2001) [29]	72/F	Left	9.5 ∗ 4.2 ∗ 4.5	Incidental finding	Open left adrenalectomy
31	Yagisawa et al. (2001) [30]	52/M	Right	6.5 ∗ 7	Dull back pain	Laparoscopic right adrenalectomy
32	Xu and Liu (2002) [31]	60/M	Right	17	Abdominal mass incidental finding	Open right adrenalectomy
33	Nursal et al. (2004) [32]	48/F	Left	13	Palpitation and unremitting hypertension	Laparotomy with left adrenalectomy
34	Wang et al. (2004) [33]	63/F	Left	5.5 ∗ 5 ∗ 4	Left upper quadrant pain	Left adrenalectomy
35	Forbes (2005) [34]	75/M	Left	19 ∗ 18 ∗ 8	Retroperitoneal hemorrhage	Laparotomy
36	Heis et al. (2008) [35]	50/F	Right	10	Flank pain	Open right adrenalectomy
37	Ng et al. (2008) [36]	59/M	Left	3.1 ∗ 2.9	Incidental finding (primary hyperaldosteronism)	Laparoscopic left adrenalectomy
38	Nigri et al. (2008) [37]	58/F	Right	7 ∗ 4.5 ∗ 3	Incidental finding	Laparoscopic right adrenalectomy
39	Arkadopoulos et al. (2009) [38]	75/F	Left	8 ∗ 6 ∗ 4	Incidental finding	Open left adrenalectomy
40	Matsuda et al. (2009) [39]	51/M	Left	4 ∗ 4 ∗ 3.5	Incidental finding	Laparoscopic left adrenalectomy
41	Siddiqi et al. (2009) [40]	54/F	Right	2.8 ∗ 2.5	Abdominal pain	NA
42	Telem et al. (2009) [41]	42/F	Left	12	Left flank pain	Laparoscopic left adrenalectomy q
43	Cheong and Kim (2010) [42]	66/F	Left	4.5 ∗ 3.4	Incidental finding	Laparoscopic left adrenalectomy
44	Paluszkieweicz et al. (2010) [43]	45/M	Left	NA	Retroperitoneal hemorrhage	Laparotomy
45	Abu EL Ghar et al. (2011) [44]	44/M	Right	11 ∗ 6	Incidental finding	NA
46	Al Jabri et al. (2011) [45]	19/F	Right	4.3 ∗ 7.3 ∗ 5.4	Incidental finding	Laparoscopic right adrenalectomy
47	kieger et al. (2011) [46]	53/F	Right	2	Microscopic hematuria	No surgical management
48	Oishi et al. (2012) [47]	75/F	Left	5 ∗ 5 ∗ 3	Incidental finding with positive metabolic workup for subclinical cushing disease	Open adrenalectomy
49	Quildrian et al. (2012) [48]	62/F	Left	12.5 ∗ 11.5 ∗ 8	Incidental finding	Open left adrenalectomy
50	Edward et al. (2013) [49]	78/F	Right	5.4 ∗ 3.3	Incidental finding	Laparoscopic right adrenalectomy
51	Galea et al. (2013) [50]	84/F	Left	13 ∗ 11	Flank pain	Open left adrenalectomy
52	Noh et al. (2014) [51]	27/F	Right	7.8 ∗ 7.8	Incidental finding	Laparoscopic right adrenalectomy
53	Wang et al. (2014) [52]	37/M	Right	6 ∗ 5 ∗ 4.5	Incidental finding	Laparoscopic right adrenalectomy
54	Agrusa et al. (2015) [53]	49/F	Right	11 ∗ 7.5 ∗ 7	Nonspecific abdominal symptoms (epigastric pain, nausea and vomiting)	Laparoscopic right adrenalectomy
55	Wong et al. (2015) [54]	80/F	Right	12.3 ∗ 13.9 ∗ 13.8	Incidental finding	Laparotomy and right adrenalectomy
56	Pang et al. (2015) [55]	71/F	Left	9.5 ∗ 8 ∗ 7.5	Chronic abdominal distention	Laparoscopic left adrenalectomy
57	Tarchouli et al. (2015) [56]	71/F	Right	42 ∗ 38 ∗ 17	Intermittent abdominal pain and increase abdominal girth	Laparotomy and open adrenalectomy
58	Bacha et al. (2016) [57]	60/M	Left	17.5 ∗ 17 ∗ 9	Incidental finding	Open adrenalectomy
59	Kinebuchi et al. (2016) [58]	77/M	Left	5.4 ∗ 4.3	Incidental finding	Laparoscopic adrenalectomy
60	Njoumi et al. (2017) [59]	30/F	Right	7	Incidental finding	Laparoscopic right adrenalectomy
61	Tadic et al. (2017) [60]	50/F	Right	11.5 ∗ 11 ∗ 11	Intermittent flank pain and abdominal discomfort	Open right adrenalectomy
62	Feo et al. (2018) [61]	70/M	Left	9 ∗ 6.5 ∗ 7	Incidental finding	Open left adrenalectomy
63	Hashimoto et al. (2018) [62]	70/M	Left	27 ∗ 17 ∗ 5.5	Loss of appetite	Laparoscopic left adrenalectomy
64	Iwamot et al. (2018) [63]	52/M	Left	5 ∗ 3.7 ∗ 3	Incidental finding	Adreno-nephrectomy
65	Lavingia et al. (2018) [64]	64/M	Right	64 ∗ 5.5 ∗ 4.7	Incidental finding	Open right adrenalectomy
66	Peng et al. (2018) [65]	31/F	Right	NA	Right upper quadrant and flank pain	Laparotomy and adrenalectomy

**Table 2:** Summary of characteristics of previously reported adrenal cavernous hemangioma in the literature.

Characteristics	Data (*N* = 66)
Median age (year)	60.04

*Sex*	
Female	41(62%)
Male	25((38%)

*Laterality*	
Right	31(47%)
Left	35(53%)
Mean size (cm)	10.8
Mean weight (g)	751.9

*Symptoms*	
Asymptomatic	38(57.5%)
Vague abdominal symptoms	8(12.1%)
Flank pain	6(9%)

*Speckled calcifications*	
Present	29(44%)
Absent	32(48.5%)

*Metabolic workup*	
Normal	45(68%)
Abnormal	6 (9%)
Hyperaldosteronism	3(4.5%)
Subclinical Cushing's syndrome	3(4.5%)

*Surgical approach*	
Open	47(71%)
Laparoscopic	16(24%)

## References

[1] Mayo-Smith W. W., Song J. H., Boland G. L. (2017). Management of incidental adrenal masses: a white paper of the ACR incidental findings committee. *Journal of the American College of Radiology*.

[2] Bhat H. S., Tiyadath B. N. (2017Mar). Management of adrenal masses. *Indian Journal of Surgical Oncology*.

[3] Johnson C. C., Jeppesen F. B. (1955 Nov). Hemangioma of the adrenal. *Journal of Urology*.

[4] Orringer R. D., Lynch J. A., McDermott W. V. (1983). Cavernous hemangioma of the adrenal gland. *Journal of Surgical Oncology*.

[5] Elliott G. B., Walker R. H., Wright A. S., Elliott K. A. (1964). Adrenal giant cyst: hemangioma of medulla with osmotic pseudocyst formation. *Annals of Surgery*.

[6] Chodoff R. J., Smith J. W., Hering N. (1966). Cavernous hemangioma of the adrenal gland. *The American Journal of Surgery*.

[7] Weiss J. M., Schulte J. W. (1966). Adrenal hemangioma: a case report. *Journal of Urology*.

[8] Ruebel A. A. (1973). Adrenal hemangioma. *Urology*.

[9] Rothberg M., Bastidas J., Mattey W. E., Bernas E. (1978). Adrenal hemangiomas: angiographic appearance of a rare tumor. *Radiology*.

[10] Vargas A. D. (1980). Adrenal hemangioma. *Urology*.

[11] Lee W. J., Weinreb J., Kumari S., Phillips G., Pochaczevsky R., Pillari G. (1982). Case report adrenal hemangioma. *Journal of Computer Assisted Tomography*.

[12] Goren E., Bensal D., Reif R. M., Eidelman A. (1986). Cavernous hemangioma of the adrenal gland. *Journal of Urology*.

[13] Nakagawa N., Takahashi M., Maeda K., Fujimura N., Yufu M. (1986). Case report: adrenal haemangioma coexisting with malignant haemangioendothelioma. *Clinical Radiology*.

[14] Guérin E., Babin C., Lehujeur C., Lucas G., Barret F. (1988). Hemangioma of the adrenal gland. Apropos of a case. *Journal of Radiology*.

[15] Derchi L. E., Rapaccini G. L., Banderali A., Danza F. M., Grillo F. (1989). Ultrasound and CT findings in two cases of hemangioma of the adrenal gland. *Journal of Computer Assisted Tomography*.

[16] Yoshihiro K., Irisawa S. (1990). Adrenal hemangioma: a case report. *Acta Urologica Japonica*.

[17] Honig S. C., Klavans M. S., Hyde C., Siroky M. B. (1991). Adrenal hemangioma: an unusual adrenal mass delineated with magnetic resonance imaging. *Journal of Urology*.

[18] Takaha N., Hosomi M., Sekii K. (1991). Retroperitoneal cavernous hemangioma: a case report. *Hinyokika Kiyo Acta Urologica Japonica*.

[19] Salup R., Finegold R., Borochovitz D., Boehnke M., Posner M. (1992). Cavernous hemangioma of the adrenal gland. *Journal of Urology*.

[20] Hamrick-Turner J. E., Grider P. L., Allen B. C., Fowler J. E., Cranston P. E., Harrison R. B. (1993). Adrenal hemangioma: MR findings with pathologic correlation. *Journal of Computer Assisted Tomography*.

[21] Sabanegh E., Harris M. J., Grider D. (1993). Cavernous adrenal hemangioma. *Urology*.

[22] Boraschi P., Campatelli A., Di Vito A., Perri G. (1995). Hemorrhage in cavernous hemangioma of the adrenal gland: US, CT and MRI appearances with pathologic correlation. * European Journal of Radiology*.

[23] Stumvoll M., Fritsche A., Wehrmann M., Dammann F., Becker H. D., Eggstein M. (1996). A functioning adrenocortical hemangioma. *Journal of Urology*.

[24] Marotti M., Sucić Z., Krolo I. (1997). Adrenal cavernous hemangioma: MRI, CT, and US appearance. *European Radiology*.

[25] Oh B. R., Jeong Y. Y., Ryu S. B., Park Y. I., Kang H. K. (1997). A case of adrenal cavernous hemangioma. *International Journal of Urology*.

[26] Hayakawa K., Sato H., Aoyagi T., Ohashi M., Ishikawa H., Hata M. (1998). Cavernous hemangioma of the adrenal gland in a patient on chronic hemodialysis. *Journal of Urology*.

[27] Hisham A. N., Samad S. A., Sharifah N. A. (1998). Huge adrenal haemangioma. *Australasian Radiology*.

[28] Makiyama K., Fukuoka H., Kawamoto K., Suwa Y. (1998). Surgical removal of adrenal hemangioma after five years of follow-up: a case report. *Hinyokika Kiyo*.

[29] Thiele J. W., Bodie B. (2001). Adrenal hemangioma.

[30] Yagisawa T., Amano H., Ito F., Horita S., Yamaguchi Y., Toma H. (2001). Adrenal hemangioma removed by a retroperitoneoscopic procedure. *International Journal of Urology*.

[31] Xu H. X., Liu G. J. (2003). Huge cavernous hemangioma of the adrenal gland: sonographic, computed tomographic, and magnetic resonance imaging findings. *Journal of Ultrasound in Medicine*.

[32] Nursal T. Z., Yildirim S., Tarim A. (2004). Giant adrenal hemangioma: a case report. *Acta Chirurgica Belgica*.

[33] Wang J. H., Chiang J. H., Chang T. (2004). Adrenal hemangioma: computed tomogram and angiogram appearances. * Chinese Medical Journal (Taipei)*.

[34] Forbes T. L. (2005). Retroperitoneal hemorrhage secondary to a ruptured cavernous hemangioma. *Canadian Journal of Surgery*.

[35] Heis H. A., Bani-Hani K. E., Bani-Hani B. K. (2008). Adrenal cavernous haemangioma. *Singapore Medical Journal*.

[36] Ng A. C., Loh H. L., Shum C. F., Yip S. K. (2008). A case of adrenal cavernous hemangioma presenting with progressive enlargement and apparent hormonal hypersecretion. *Endocrine Practice*.

[37] Nigri G., Bellagamba R., Giaccaglia V. (2008). Minimally invasive adrenalectomy for incidentally discovered cavernous hemangioma. *Minimally Invasive Therapy & Allied Technologies*.

[38] Arkadopoulos N., Kyriazi M., Yiallourou A. I. (2009). A rare coexistence of adrenal cavernous hemangioma with extramedullar hemopoietic tissue: a case report and brief review of the literature. *World Journal of Surgical Oncology*.

[39] Matsuda D., Iwamura M., Baba S. (2009). Cavernous hemangioma of the adrenal gland. *International Journal of Urology*.

[40] Siddiqi A. J., Miller F. H., Kasuganti D., Nikolaidis P. (2009). Adrenal hemangioma-adenoma: an exceedingly rare adrenal collision tumor. * Journal of Magnetic Resonance Imaging*.

[41] Telem D. A., Nguyen S. Q., Chin E. H., Weber K., Divino C. M. (2009). Laparoscopic resection of giant adrenal cavernous hemangioma. *Journal of the Society of Laparoendoscopic Surgeons*.

[42] Cheong J. H., Kim G. H. (2010). A case of adrenal hemangioma misdiagnosed as a pancreatic tail tumor. *The Korean Journal of Gastroenterology*.

[43] Paluszkiewicz P., Ambroziak I., Hołyńska-Dąbrowska K., Siezieniewska-Skowrońska Z., Paluszkiewicz A. (2010). Spontaneous rupture of adrenal haemangioma mimicking abdominal aortic aneurysm rupture. *Archives of Medical Science*.

[44] Abou El-Ghar M., Refaie H., El-Hefnawy A., El-Diasty T. (2011). Adrenal hemangioma: findings at multidetector CT with short review of the literature. *Case Reports in Radiology*.

[45] Aljabri K. S., Bokhari S. A., Alkeraithi M. (2011). Adrenal hemangioma in a 19-year-old female. *Annals of Saudi Medicine*.

[46] Kieger A. J., Nikolaidis P., Casalino D. D. (2011). Adrenal gland hemangioma. *Journal of Urology*.

[47] Oishi M., Ueda S., Honjo S., Koshiyama H., Yuba Y., Takabayashi A. (2012). Adrenal cavernous hemangioma with subclinical Cushing’s syndrome: report of a case. *Surgery Today*.

[48] Quildrian S. D., Silberman E. A., Vigovich F. A., Porto E. A. (2013). Giant cavernous hemangioma of the adrenal gland. *International Journal of Surgery Case Reports*.

[49] Edwards J. P., Stuart H. C., Urbanski S. J., Pasieka J. L. (2014). A rare cavernous hemangioma of the adrenal gland. * International Journal of Surgery Case Reports*.

[50] Galea N., Noce V., Ciolina F., Liberali S., Francone M. (2013). Giant adrenal cavernous hemangioma: a rare abdominal mass. *Urology*.

[51] Noh J. J., Choi S. H., Hwang H. K., Kang C. M., Lee W. J. (2014). Adrenal cavernous hemangioma: a case report with review of the literature. *Journal of Periodontology*.

[52] Wang L., Dang Y., He R., Chen G. (2014). Rare cavernous hemangioma of adrenal gland: case report. *Sao Paulo Medical Journal*.

[53] Agrusa A., Romano G., Salamone G. (2015). Large cavernous hemangioma of the adrenal gland: laparoscopic treatment. Report of a case. * International Journal of Surgery Case Reports*.

[54] Wong G. L., Kwok R., Wong V. W. (2015). Huge adrenal hemangioma: a rare cause of deceivingly high liver stiffness measurement by transient elastography. *Clinical Gastroenterology and Hepatology*.

[55] Pang C., Wu P., Zhu G. (2015). A rare cavernous hemangioma of the adrenal gland. * Urology Case Reports*.

[56] Tarchouli M., Boudhas A., Ratbi M. B. (2015). Giant adrenal hemangioma: unusual cause of huge abdominal mass. * Canadian Urological Association Journal*.

[57] Bacha D., Chaabane A., Khanche F., Néchi S., Touinsi H., Chelbi E. (2016). Giant adrenal cavernous hemangioma in a patient with familial adenomatous polyposis. *Clinics and Practice*.

[58] Kinebuchi Y., Daimon H., Kawaguchi K. (2016). Adrenal cavernous hemangioma associated with myelolipomatous metaplasia. *International Journal of Urology*.

[59] Njoumi N., Jakhlal N., Laaroussi M. (2017). Adrenal gland hemangioma: about a case. *Pan African Medical Journal*.

[60] Tadić B., Grubor N., Milosavljević V., Matić S., Grubor N., Ignjatovic I. (2017). Giant cavernous hemangioma of the adrenal gland: case report and review of the literature. *Journal of Clinical Case Reports*.

[61] Feo C. V., De Troia A., Pedriali M. (2018). Adrenal cavernous hemangioma: a case report. *BMC Surgery*.

[62] Hashimoto A., Yoshino H., Yoshikawa F. (2018). giant cavernous hemangioma of the adrenal gland in an elderly patient. *Internal Medicine*.

[63] Iwamoto G., Shimokihara K., Kawahara T. (2018). Adrenal hemangioma: a case of retroperitoneal tumor. *Case Reports in Medicine*.

[64] Lavingia K., Torabi R., Kim S. W., Hughes M. S., Feliberti E. C., Perry R. R. (2018). A rare adrenal incidentaloma that mimics adrenocortical carcinoma. *Case Reports in Surgery*.

[65] Peng X., Luo W., Zhang X., Zhu W. (2018). Sudden onset flank pain: a case report of retroperitoneal hemorrhage secondary to a ruptured adrenal hemangioma. *Journal of Pain Research*.

[66] Yamada T., Ishibashi T., Majima Saito H. K, Tsuda M., Takahashi S., Moriya T. (2002). Two cases of adrenal hemangioma: CT and MRI findings with pathological correlations. *Radiation Medicine*.

[67] Krebs T. L., Wagner B. J. (1998). MR imaging of the adrenal gland: radiologic pathologic correlation. *Radiographics*.

[68] Ishigami K., Stolpen A. H., Sato Y., Dahmoush L., Winfield H. N., Fajardo L. L. (2004). Adrenal adenoma with organizing hematoma:diagnostic dilemma at MRI. *Magnetic Resonance Imaging*.

[69] Quint L. E., Glazer G. M., Francis I. R., Shapiro B., Chenevert T. L. (1987). Pheochromocytoma and paraganglioma: comparison of MR imaging with CT and I-131 MIBG scintigraphy. *Radiology*.

[70] Elfenbein D. M., Scarborough J. E., Speicher P. J., Scheri R. P. (2013). Comparison of laparoscopic versus open adrenalectomy: results from American College of Surgeons-National Surgery Quality Improvement Project. *Journal of Surgical Research*.

